# Robust high-throughput prokaryote *de novo* assembly and improvement pipeline for Illumina data

**DOI:** 10.1099/mgen.0.000083

**Published:** 2016-08-25

**Authors:** Andrew J. Page, Nishadi De Silva, Martin Hunt, Michael A. Quail, Julian Parkhill, Simon R. Harris, Thomas D. Otto, Jacqueline A. Keane

**Affiliations:** ^1^​ Pathogen Informatics, Wellcome Trust Sanger Institute, Wellcome Genome Campus, Hinxton, CB10 1SA, Cambridgeshire, UK; ^2^​ Biochemical Development, Wellcome Trust Sanger Institute, Wellcome Genome Campus, Hinxton, CB10 1SA, Cambridgeshire, UK; ^3^​ Pathogen Genomics, Wellcome Trust Sanger Institute, Wellcome Genome Campus, Hinxton, CB10 1SA, Cambridgeshire, UK; ^4^​ Parasite Genomics, Wellcome Trust Sanger Institute, Wellcome Genome Campus, Hinxton, CB10 1SA, Cambridgeshire, UK

**Keywords:** illumina, assembly, high-throughput, prokaryotic

## Abstract

The rapidly reducing cost of bacterial genome sequencing has lead to its routine use in large-scale microbial analysis. Though mapping approaches can be used to find differences relative to the reference, many bacteria are subject to constant evolutionary pressures resulting in events such as the loss and gain of mobile genetic elements, horizontal gene transfer through recombination and genomic rearrangements. *De novo* assembly is the reconstruction of the underlying genome sequence, an essential step to understanding bacterial genome diversity. Here we present a high-throughput bacterial assembly and improvement pipeline that has been used to generate nearly 20 000 annotated draft genome assemblies in public databases. We demonstrate its performance on a public data set of 9404 genomes. We find all the genes used in multi-locus sequence typing schema present in 99.6 % of assembled genomes. When tested on low-, neutral- and high-GC organisms, more than 94 % of genes were present and completely intact. The pipeline has been proven to be scalable and robust with a wide variety of datasets without requiring human intervention. All of the software is available on GitHub under the GNU GPL open source license.

## Data Summary

1. The optional assembly pipeline software is available from Github under the GNU GPL open source license; (url – https://github.com/sanger-pathogens/vr-codebase)
2. The assembly improvement software is available from Github under the GNU GPL open source license; (url – https://github.com/sanger-pathogens/assembly_improvement)
3. Accession numbers for 9404 assemblies are provided in the supplementary material.
4. The *Bordetella pertussis* sample has sample accession ERS1058649, sequencing reads accession number ERR1274624 and assembly accession numbers FJMX01000001-FJMX01000249; (url – http://www.ebi.ac.uk/ena/data/view/ERR1274624)
5. The *Salmonella enterica *subsp.* enterica *serovar Pullorum sample has sample accession number ERS1058652, sequencing reads accession number ERR1274625 and assembly accession numbers FJMV01000001–FJMV01000026; (url – http://www.ebi.ac.uk/ena/data/view/ERR1274625)
6. The *Staphylococcus aureus* sample has sample accession number ERS1058648, sequencing reads accession number ERR1274626 and assembly accession numbers FJMW01000001–FJMW01000040; (url – http://www.ebi.ac.uk/ena/data/view/ERR1274625)

## Impact Statement

The automated generation of *de novo* assemblies is a critical step in exploring bacterial genome diversity. The pipeline described in this paper has been used to assemble and annotate 30 % of all bacterial genome assemblies in GenBank (18 080 out of 59 536, accessed 16/2/16). Rather than being optimized for the highest quality assembly, it is optimised for efficient resource usage, throughput and robustness. Multi-locus sequence typing genes are found in 99.6 % of cases, making it at least as good as existing typing methods. In the test genomes we present, more than 94 % of genes are correctly assembled into intact reading frames.

## Introduction

The rapid reduction in the cost of whole-genome sequencing (WGS) has made it feasible to sequence thousands of prokaryotic samples within a single study (Chewapreecha *et al.*, 2014; Nasser *et al.*, 2014; Wong *et al.*, 2015). Many bacteria acquire genetic material through horizontal gene transfer when different strains recombine (Croucher *et al.*, 2011). Mobile genetic elements such as phages, plasmids and transposons, by their very nature, are the most variable part of the genome, enabling rapid exchange of genetic material between isolates. They are known to carry antibiotic resistance and virulence genes, and so are some of the most biologically interesting parts of the genome (Medini *et al.*, 2005). Identification of lost sequences and genes is also biologically important as this can signal host or environment adaptation (Klemm *et al.*, 2016). Though reconstructing the sequence (*de novo* assembly) and performing annotation is a more complex process than performing a mapping-based approach, it will: (1) generate sequences not in the reference genome [variable accessory genome (Page *et al.*, 2015)], (2) resolve deletions which generate errors in mapping-based approaches, (3) find signatures of recombination (Croucher *et al.*, 2014), and (4) enable the community to work with a full sequence for bottom-up analysis from public databases, rather than single-nucleotide polymorphism lists.

Although *de novo* assembly is computationally challenging (Pop, 2009) it has many advantages over mapping-based approaches. One of the fundamental limitations of *de novo* assembly is that any repetitive regions within the genome that exceed the length of the library fragment size prevent a complete *de novo* assembly from paired-end reads. However, the most cost effective, and hence most common, sequencing method involves sequencing the ends of short DNA fragments (<1000 bp). When a repeat region is larger than the fragment size, the assembler cannot unambiguously reconstruct the underlying sequence, so a break is introduced. This challenge has been addressed in a number of different ways. Automatically tuning parameters and configurations can produce improved assemblies, such as using RAMPART (Mapleson *et al.*, 2015). The iMetAMOS pipeline (Koren *et al.*, 2014) uses multiple different assemblers and picks the best result, however it takes on average over one month to assemble a single bacterial genome, which makes it computationally unfeasible to run on a large number of samples. Assemblies may be improved using wet lab methods (Puranik *et al.*, 2015), such as using capilary sequencing to extend over gaps, optical mapping, or additional long-insert mate-pair libraries, however these approaches are low-throughput and prohibitively costly. Several tools mirror these manual steps, like ordering contigs (Assefa *et al.*, 2009), performing scaffolding (Hunt *et al.*, 2014), automating gap closing (Tsai *et al.*, 2010; Boetzer & Pirovano, 2012; Walker *et al.*, 2014), correcting base errors (Otto *et al.*, 2010; Walker *et al.*, 2014) and assembly error identification (Hunt *et al.*, 2013). Some tools implement a collection of these steps, such as SPAdes (Bankevich *et al.*, 2012), MaSuRCA (Zimin *et al.*, 2013) and iMetAMOS (Koren *et al.*, 2014), but these additional steps come with computational overheads which can substantially increase the overall running time. In some cases it is desireable to produce the highest quality genomes using manual and automated methods, for example when generating a new reference genome for a species. However, in a lot of cases, a draft genome will contain enough of the sequences and genes to allow useful analysis to be performed (Wong *et al.*, 2015; Makendi *et al.*, 2016; Page *et al.*, 2015) without the computational overheads.

The annotation of bacterial genomes can be performed using a number of automated tools (Seemann, 2014; Mitchell *et al.*, 2015). Although we have seen a commoditisation of sequencing technologies due to rapidly decreased costs, the generation of annotated genomes, and deposition of those to the public archives (EMBL/GenBank), can be a very time consuming and laborous process, so is rarely performed (Pirovano *et al.*, 2015). Taking the *Salmonella* genus as an example: of the 44 920 WGS samples sequenced, only 4451 (9.9 %) have had assemblies depositied in GenBank (accessed 5 May 2016).

To overcome these challenges, whilst also balancing computational overhead and robustness versus quality, we have created a reliable assembly and improvement pipeline that consistently produces annotated genomes on a large scale ready for uploading to EMBL/EBI. To date, 18 080 *de novo* assemblies have been created and submitted to public databases, with associated epidemiological metadata, from 10 Tbp of raw sequencing data. The pipeline is robust to failure, auto restarting when one step fails. It estimates the amount of memory required. It performs multiple assemblies and several automated *in-silico* improvement steps that increase the contiguity of the resulting assembly. We assess the quality of the assemblies for low-, neutral- and high-GC genomes. The pipeline is written in Perl and is freely available under the open source GNU GPL license.

## Theory and implementation

**Fig. 1.**
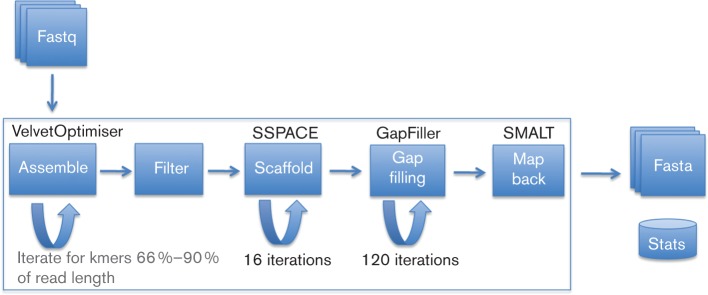
Overview of the method with major components noted.

An overview of the method is shown in Fig. 1 and a step-by-step example is available from (Page, 2016a). For each genome, the *de novo* short-read assembler Velvet (Zerbino, 2010) is used to generate multiple assemblies by varying the k-mer size between 66 % and 90 % of the read length using VelvetOptimiser (Gladman & Seemann, 2008), as a well-chosen k-mer can substantially increase the quality of the resulting assembly (Zerbino, 2010). These assemblies can be optionally run through a pipeline system (Bala *et al.*, 2016). From these assemblies, the assembly with the highest N50 is chosen. The N50 is the length *L* of the longest contig such that half of the nucleotides in the assembly lie in contigs of length at least *L.* When the pipeline was initially written (2012), the Velvet assembler was chosen because it proved to be robust to a wide range of data sets during testing and had a low computational overhead (Abbas *et al.*, 2014) compared with SPAdes (Bankevich *et al.*, 2012). Comparisons with the current version of SPAdes (v3.8.0) show similar performance and quality of results to Velvet and are detailed in Table S1 (available in the online Supplementary Material).

A stand-alone assembly improvement step is run on the assembly to scaffold the contigs using SSPACE (Boetzer *et al.*, 2011) and fill in sequence gaps using GapFiller (Boetzer & Pirovano, 2012). First, to reduce the computational burden, reads that map [SMALT (Ponstingl & Ning, 2015)] as proper pairs are excluded, since they have been successfully used in the assembly. A proper pair is a pair of reads from the same fragment of DNA which align to a single contig, in the correct orientation, within the expected insertion size range. The remaining reads, which are either unmapped or are mapped but have a mate that was unmapped or mapped to a different contig, are used for the improvement step. As SSPACE and GapFiller are greedy algorithms, they make assumptions, which can lead to false joins. We control for this by iteratively lowering the read coverage required to make a join, so that contigs with the most read-pair evidence are joined in an earlier iteration than contigs with low read-pair evidence. As we use a subset of reads (those not mapping in perfect pairs to a contig), this step is very fast, requiring between 20 and 60 min (Table S2, available in the online Supplementary Material) for a single sample.

On the first iteration a minimum of 90 read pairs must link two contigs for them to be joined. This is then progressively reduced over 16 iterations down to five read pairs. These parameters were chosen after extensive testing on a range of organisms. Where two contigs are joined by read pairs, a gap consisting of an unknown number of bases (N) is generated. These gaps are targeted for closure by running 120 iterations of GapFiller (Boetzer & Pirovano, 2012) (version 1.11), using a similar decreasing read evidence threshold beginning with a minimum depth of coverage of 90 reads, alternating between BWA (Li & Durbin, 2009) and Bowtie (Langmead *et al.*, 2009). Contigs are excluded from the assembly where they are shorter than the target fragment size (normally 300–500 bases). The contigs are then sorted by size and renamed in a standardised manner to include the raw sequencing data accession number. Finally, to assess the quality of the assembly and to produce a set of statistics, the reads are aligned again to the final assembly using SMALT. All the assemblies produced are created in a standardised manner and require no input from the user. The assemblies are then automatically annotated using PROKKA (Seemann, 2014) with genus-specific databases from RefSeq (Pruitt *et al.*, 2012). The resulting annotated assemblies are in a format suitable for submission to EMBL/GenBank with post processing using GFF3toEMBL (Page *et al.*, 2016). All the assemblies produced are created in a standardised manner and require no input from the user.

To assess the quality of the assemblies produced by the pipeline we used three microbial genomes with differing G+C content: *Bordetella pertussis *(67 %), *Salmonella enterica *subsp.* enterica *serovar Pullorum (52 %) and *Staphylococcus aureus *(33 %). This is a standard set of strains used for technology validation at the Wellcome Trust Sanger Institute (Quail *et al.*, 2012). A closed complete capillary reference genome is available for each, with the *Salmonella enterica *subsp.* enterica *serovar Pullorum and *Staphylococcus aureus *TW20 (Holden *et al.*, 2010) data originating from the same isolate. Each genome was paired-end sequenced on the Illumina MiSeq with a read length of 130 bp, achieving a coverage of 28–43×. We compared the pipeline assemblies in each case to the capillary reference genomes using QUAST (Gurevich *et al.*, 2013) and present the results in Table 1. Overall the assemblies contained at least 94 % of the reference genome, so are good representations of the underlying genome. *Salmonella enterica *subsp.* enterica *serovar Pullorum was assembled into 22 contigs and *Staphylococcus aureus *into 38 contigs*. B. pertussis* is known to contain many repetitive IS elements, explaining the higher level of fragmentation, which at 247 contigs is approximately equal to the number of IS elements (261 out of 3816 genes in *B. pertussis* Tohama I annotated as IS elements). A pan genome was constructed using Roary (Page *et al.*, 2015) for each organism, consisting of the predicted genes (Seemann, 2014) from the reference and *de novo* assembly. The *de novo* assemblies contained 93–98 % of the reference genes. This is in agreement with the percentage of the nucleotide bases matching between the *de novo* assembly and the reference, but does not account for misassemblies.

**Table 1.** Comparison of *de novo* assemblies derived from the pipeline against their corresponding complete reference genomes using QUAST More comprehensive details are available in Table S1.

Organism	*B. pertussis*	*S.* *enterica* subsp. *enterica *s*erovar* Pullorum	*S. aureus*
Coverage	40.16	28.11	43.86
Number of contigs	247	22	38
Total length	3 856 742	4 711 864	3 016 231
Reference length	4 086 189	4 895 678	3 075 806
Genome fraction (%)	94.32	95.74	98.00
DNA GC content (%)	67.81	52.15	32.64
Reference DNA GC content (%)	67.72	52.16	32.78
N50	23 177	517 904	206 505
Number of misassemblies	6	10	4
Number of mismatches per 100 kbp	1.43	1.15	1.76
Number of indels per 100 kbp	0.6	1.92	0.17
Genes	3624	4727	2965
Percentage of reference genes found	93.19	95.19	98.41

**Fig. 2.**
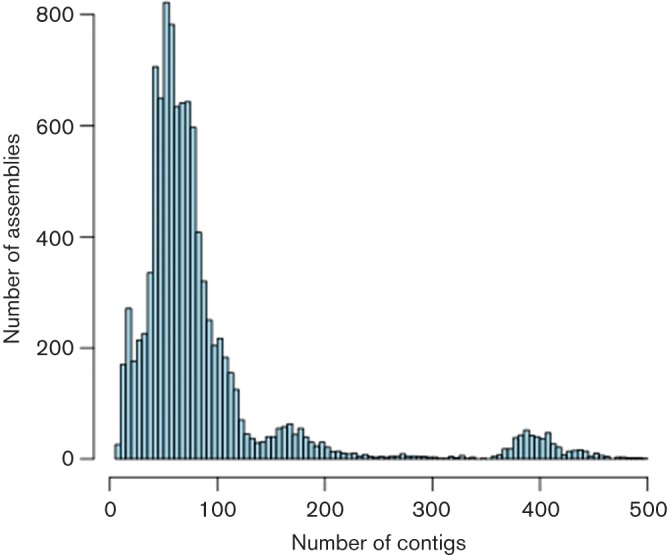
Distribution of the number of contigs in a set of 9404 assemblies.

**Fig. 3.**
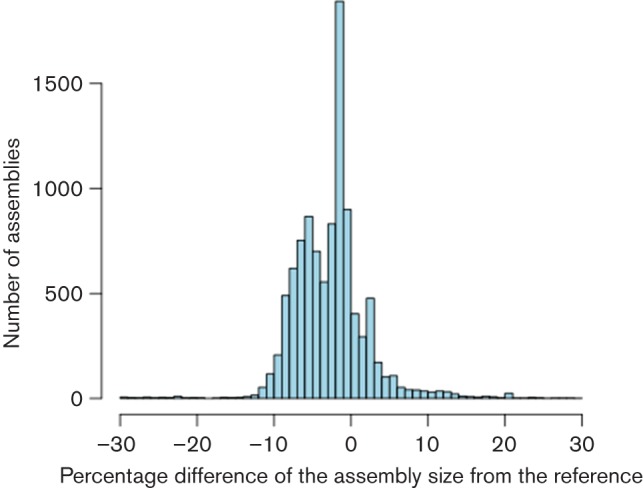
Distribution of the percentage difference between the size of each assembly and the size of a closely related reference sequence.

To assess the performance of the pipeline on a large scale we took a set of 18 080 published public assemblies and filtered them down to a set of 9404 assemblies covering 73 bacterial species, summarised in Table 2. Only assemblies from isolates sequenced at the Wellcome Trust Sanger Institute on the Illumina HiSeq 2000/2500 or MiSeq platforms to high coverage (>50×) were considered. Contaminated samples were excluded after taxonomic classification of the raw reads with Kraken (Wood & Salzberg, 2014) and where more than one multi-locus sequence typing (MLST) allele was found (37 assemblies). Fig. 2 gives the distribution of the number of contigs in each assembly. The mean is 89 contigs with peaks corresponding to different species, such as *Shigella* at 405 contigs. Before an isolate is sequenced, a reference genome is chosen based on the predicted species. We compared the size of the assembly to the size of the corresponding reference and present the distribution in Fig. 3. Of all assemblies, 98 % are within ±10 % of the size of their corresponding reference genome. Some natural variation is to be expected within bacteria, for example the size of *Escherichia coli *genomes can vary by more than 20 % (Blattner *et al.*, 1997; Perna *et al.*, 2001). Some may be larger because of plasmids or phages; others may have experienced gene loss and are smaller. However, most of the assemblies are at the expected size, allowing for useful comparisons to be made (Wong *et al.*, 2015; Makendi *et al.*, 2016; Page *et al.*, 2015).

**Table 2.** Summary of the isolates in the large public dataset

Species	Number of samples	Mean contigs	Mean coverage
*Burkholderia pseudomallei*	168	70	134
*Campylobacter jejuni*	379	24	121
*Escherichia coli*	178	167	145
*Mycobacterium abscessus*	157	37	120
*Mycobacterium tuberculosis*	1441	122	150
*Neisseria gonorrhoeae*	234	75	205
*Salmonella enterica*	1643	55	92
*Salmonella *Typhimurium	171	81	136
*Shigella sonnei*	299	405	118
*Staphylococcus aureus*	534	36	174
*Staphylococcus haemolyticus*	131	86	91
*Streptococcus agalactiae*	116	26	293
*Streptococcus equi*	159	81	374
*Streptococcus pneumoniae*	3562	74	290
Other	232	80	136

Seven gene MLST schemes based on essential housekeeping genes exist for 6971 of the assemblies (Maiden *et al.*, 1998) from the set of 9404 assemblies. These sequence-typing methods are widely used by reference labs for genomic epidemiology, predating whole-genome sequencing technologies. If all of the MLST genes are present in the assemblies then it allows for the assemblies to be used as a replacement for traditional PCR-based methods. The MLST scheme for *Mycobacterium abscessus* is poorly populated, containing very few alleles and we could only assign an allele in 30 % of cases, so has been excluded from this analysis, leaving 6814 assemblies. Only genes with at least 95 % length and identity to a known MLST allele are counted as a match. We found that in 6789 (99.6 %) assemblies we could identify all of the MLST genes using MLST-check (Page, 2016b), a method which performs a nucleotide blast (Camacho *et al.*, 2009) of all the MLST alleles against each assembly, with the latest databases downloaded from pubMLST (Jolley & Maiden, 2010). One MLST gene was missing from each of 16 assemblies (0.23 %). One sample (0.013 %) was only partially assembled but on closer investigation it had unusually high coverage (445×), which appears to have lead to a poor choice of *k*-mer. Of the remaining eight assemblies, where the sequence type could not be inferred from the assembly, all contained contaminations that were identified as different species when analysed with Kraken (Wood & Salzberg, 2014).

## Conclusion

Generating annotated genomes from whole-genome sequencing data is a complex and laborious process that enables the true diversity within a species to be unveiled. We developed a high-throughput pipeline that has been used to generate 30 % of all bacterial assemblies in GenBank. The resulting genomes encompass more than 94 % of the predicted genes and nucleotides, and have MLST genes available in 99.6 % of assembled samples over a range of organisms with different DNA GC contents. We demonstrate that it has been successfully scaled up to tens of thousands of samples, providing annotated *de novo* assemblies suitable for submission to EMBL/GenBank without the need for manual intervention.

## Supplementary Data

1658 Supplementary File 1

## Supplementary Data

246 Supplementary File 2
