# Supplementary material for: Robust high-throughput prokaryote de novo assembly and improvement pipeline for Illumina data
Source: Microb Genom. 2016 Aug 25;2(8):e000083. doi: 10.1099/mgen.0.000083 (PMC5320598; doi:10.1099/mgen.0.000083)
Supplement: Supplementary file 2 [file mgen-02-83-s002.docx]

Supplementary Material for Robust high throughput prokaryote *de novo* assembly and improvement pipeline for Illumina data

# : SPAdes kmers evaluation

## Sample data

To compare the spades and velvet assemblers and assess the different assembly assessment metrics, we used three microbial genomes with differing G+C content: Bordetella *pertussis* (67%) (ERR1274624), Salmonella Pullorum (52%) (ERR1274625) and Staphylococcus *aureus* (33%) (ERR1274626).

## Spades assemblies

For each of the 3 samples, we generated assemblies using the SPAdes assembler, version 3.8.0 (release date 31 May 2016). We used the default settings in addition to the same range of kmer values provided to Velvet to allow for a fair comparison. We present experimental results in the next section to support using these additional kmer values.

## Choosing kmer values for SPAdes

For each of the 3 samples in the paper (listed in Section 1.01) we ran SPAdes with a range of kmer values. By default SPAdes assembles using kmers of lengths 21, 33, and 55 and chooses the assembly with the best N50 score. As our reads were 130 bases in length, the maximum kmer of 55 was thought to be too small. To test this we ran SPAdes with an expanded range of kmers: 21, 33, 55, 61, 71, 81, 91, 101, 111, and 121. Supplementary Figure 1 shows that as the kmer varies for each sample assembly the N50 varies with some of the larger kmer values producing larger N50 than than the defaults. There is a subset of kmers between 55 and 101 where the N50 is maximized in all samples, with a drop off on either end. Only 55 is used by SPAdes as part of its default kmer set, and it gives a smaller N50 in the *S*. Pullorum sample. N50 alone does not give the full picture of the quality of an assembly, however it is a commonly used metric. Supplementary Figure 2 shows the number of contigs for each assembly with varying kmers (a smaller number of contigs is desirable). Using only the default kmers would have resulted in a highly fragmented *S*. Pullorum assembly with 1747 contigs compared to a kmer size of 101 which resulted in 64 contigs. The best overall performance for this metric was with kmers of size 91 and 101. Finally, Supplementary Figure 3 shows the overall total length of the resulting assemblies as the kmers vary. For *S. aureus* and *B. pertussis* the total length is consistent with the expected genome length over all kmers (except 121). *S*. Pullorum however contains substantial amounts of extra content for low kmers. Further analysis reveals that there are over 1000 very short contigs containing duplicated sequence, which map throughout the genome. Increasing the kmer length for S. Pullorum to between 91 and 111 brought the assembly inline with the expected length (4.7 Mb). Using the default SPAdes kmers in this instance would have resulted in 500 Kbases of duplicated sequences, complicating further analysis. As a result of these experiments we decided that the default kmers used by SPAdes would be unsuitable for this dataset of low, neutral and high GC bacteria.

Supplementary Figure 1: Assembly N50 with varying kmers using SPAdes

Supplementary Figure 2: Number of contigs in each SPAdes assembly as the kmer is varied.

Supplementary Figure 3: Total length in bases of each SPAdes assembly as the kmer is varied.

# Comparison of Velvet and SPAdes

When the pipeline was initially developed in 2012, SPAdes was in the early stages of development which made it unsuitable for use on a large scale (high computational costs, memory, and under rapid development). Velvet on the other hand was stable, robust, and used resources efficiently. Since then, SPAdes has been further developed and improved For comparison, we substituted the latest version of SPAdes (v3.8.0) in place of VelvetOptimiser. Based on our analysis of the kmer range for SPAdes (see Section 1.01) we provided the same set of kmers to SPAdes as was used by VelvetOptimiser (which included the default set used by SPAdes), using every odd number between 87 and 117 (66%-90% of the read length).

The 3 samples were assembled using all kmers as described above, using SPAdes (v3.8.0) and the results are given in Supplementary Table 1. The total running time for both SPAdes and Velvet is comparable, averaging between 5-9 hours for a single bacteria using a single CPU.

What happened next, the assemblies with the best N50 were chosen and evaluated using QUAST v3.2. The statistics are very similar between SPAdes and VelvetOptimiser, with SPAdes producing marginally better results. The assembly with the highest N50 was chosen by SPAdes. The number of genes correctly assembled is similar in both assemblies, with the exception of S. Pullorum, where SPAdes correctly assembles an additional 1% of genes.

|  | **B. pertussis**  **ERR1274624** | | **S. Pullorum**  **ERR1274625** | | **S. aureus**  **ERR1274626** | |
| --- | --- | --- | --- | --- | --- | --- |
|  | **VelvetOpt** | **Spades** | **VelvetOpt** | **Spades** | **VelvetOpt** | **Spades** |
| **Assembly cpu time (h)** | 01:15:00 | 01:52:22 | 00:52:16 | 01:33:22 | 01:00:22 | 01:11:22 |
| **Improvement (h)** | 01:03:03 | 00:58:10 | 00:56:35 | 00:19:42 | 00:25:23 | 00:20:09 |
| **Map back (h)** | 00:46:43 | 00:45:54 | 00:16:49 | 00:14:14 | 00:19:08 | 00:11:04 |
| **Annotation (h)** | 05:13:27 | 05:27:16 | 05:34:18 | 04:28:02 | 04:13:45 | 03:43:17 |
| **Total (h)** | 08:18:13 | 09:03:42 | 07:39:58 | 06:35:20 | 05:58:38 | 05:25:52 |
|  |  |  |  |  |  |  |
| **Assembly RAM (GB)** | 1.4 | 1.2 | 1.5 | 1.1 | 1.3 | 1.1 |
| **Map back (GB)** | 0.5 | 0.5 | 0.4 | 0.4 | 0.4 | 0.4 |
| **Annotation (GB)** | 0.3 | 0.3 | 0.5 | 0.5 | 3.3 | 3.4 |
| **Peak RAM (GB)** | 1.4 | 1.2 | 1.5 | 1.1 | 3.3 | 3.4 |
|  |  |  |  |  |  |  |
| **No. of contigs** | 247 | 246 | 22 | 20 | 38 | 35 |
| **Total length** | 3856742 | 3892592 | 4711864 | 4710659 | 3016231 | 3016948 |
| **Reference length** | 4086189 | 4086189 | 4895678 | 4895678 | 3075806 | 3075806 |
| **Genome fraction (%)** | 94.323 | 95.231 | 95.744 | 96.142 | 97.996 | 97.964 |
| **GC (%)** | 67.81 | 67.77 | 52.15 | 52.17 | 32.64 | 32.64 |
| **Reference GC (%)** | 67.72 | 67.72 | 52.16 | 52.16 | 32.78 | 32.78 |
| **N50** | 23177 | 23177 | 517904 | 642692 | 206505 | 490731 |
| **Misassemblies** | 6 | 5 | 10 | 9 | 4 | 5 |
| **Mismatches/100 kbp** | 1.43 | 3.21 | 1.15 | 2.97 | 1.76 | 3.09 |
| **Indels/100 kbp** | 0.6 | 0.54 | 1.92 | 0.49 | 0.17 | 0.17 |
| **Genes** | 3624 | 3833 | 4727 | 4744 | 2965 | 2964 |
| **% reference genes found** | 93.19 | 93.25 | 95.19 | 96.16 | 98.41 | 98.34 |

Supplementary Table 1: Comparison of VelvetOptimiser and SPAdes assemblies with: running time, memory and assembly metrics.

# Evaluating VelvetOptimiser assemblies with other assembly metrics

## VelvetOptimiser assemblies

Each of the 3 samples were assembled *de novo* using VelvetOptimiser. Each of the intermediate assemblies produced by VelvetOptimiser for each kmer were retained for evaluation and no post improvement step was run on these assemblies. This allowed us to evaluate which assembly would have been chosen by VelvetOptimiser based on N50 and which assembly would have been chosen if CGAL or LAP were used.

Supplementary Figure 4 shows that the best N50 is found at the beginning of the range of kmers. Supplementary Figure 5 also shows that the lowest number of contigs (lower is better) is also found in this range, and Supplementary Figure 6 shows that the expected total size of the underlying assemblies is mostly consistent across a wide range of kmers, with the exception of the largest.

Supplementary Figure 4: N50 of each Velvet assembly.

##

Supplementary Figure 5: Number of contigs for each Velvet assembly.

##

Supplementary Figure 6: Total combined length of the sequences of each Velvet assembly in bases.

## CGAL

Each of the intermediate assemblies produced by VelvetOptimiser were assessed using CGAL (version 0.9.6-beta) (Rahman & Pachter 2013) with the procedure and parameters recommended by the authors. They were first aligned with bowtie2 (version 2.1.0) using the parameters required by CGAL (-a --no-mixed). The total running time for evaluating a single assembly using a single CPU exceeded 2 days in all cases, after which the processes were terminated as any time beyond this was deemed to be infeasible to run on a large scale.

## LAP

Each of the intermediate assemblies produced by VelvetOptimiser were evaluated with LAP (version 1.1) using the parameters recommended by the authors (Ghodsi et al. 2013). For each of the assemblies, the best LAP (larger) score is to be found at the lower range of kmers.

Supplementary Figure 7: The LAP score of each Velvet assembly with varying kmers.

## Comparing assembly metrics

Supplementary Table 2 shows the running time of each of the assembly metrics, and is the sum total of computing the metric for all of the kmers for a sample. The LAP metric identified the lower kmers as better. The assemblies chosen based on N50 alone also fall into this range of kmers, however the computational cost to calculate the LAP score is much higher than calculating the N50 (under 1 second versus hours). The total running time of LAP also varies greatly from ~1.5 hours up to ~4 hours. The computational cost of CGAL exceeded many days which we deem to be unsuitable for running on a large scale, however it may be reasonable in the case of a single high quality reference genome, where the extra computational cost can be justified.

|  | N50 (seconds) | LAP (seconds) | CGAL |
| --- | --- | --- | --- |
| B. pertussis | < 1 | 14292 | Over 2 days |
| S. aureus | < 1 | 7020 | Over 2 days |
| S. Pullorum | < 1 | 5796 | Over 2 days |

Supplementary Table 2: Time in seconds to calculate assembly metrics. This is the combined total for running the metric over all of the assemblies produced by VelvetOptimiser using a single CPU. The CGAL processes were terminated without finishing after 2 days of computation.

# References

Ghodsi, M. et al., 2013. De novo likelihood-based measures for comparing genome assemblies. *BMC Research Notes*, 6, p.334. Available at: http://www.ncbi.nlm.nih.gov/pmc/articles/PMC3765854/.

Rahman, A. & Pachter, L., 2013. CGAL: computing genome assembly likelihoods. *Genome Biol*, 14(1), p.R8. Available at: http://www.pubmedcentral.nih.gov/articlerender.fcgi?artid=3663106&tool=pmcentrez&rendertype=abstract.
